# Light Sources in Hyperspectral Imaging Simultaneously Influence Object Detection Performance and Vase Life of Cut Roses

**DOI:** 10.3390/plants15020215

**Published:** 2026-01-09

**Authors:** Yong-Tae Kim, Ji Yeong Ham, Byung-Chun In

**Affiliations:** Department of Smart Horticultural Science, Gyeongkuk National University, Andong 36729, Republic of Korea; qkfkadpc@gknu.ac.kr (Y.-T.K.);

**Keywords:** deep learning, hyperspectral camera, imaging, object detection, cut roses, vase life

## Abstract

Hyperspectral imaging (HSI) is a noncontact camera-based technique that enables deep learning models to learn various plant conditions by detecting light reflectance under illumination. In this study, we investigated the effects of four light sources—halogen (HAL), incandescent (INC), fluorescent (FLU), and light-emitting diodes (LED)—on the quality of spectral images and the vase life (VL) of cut roses, which are vulnerable to abiotic stresses. Cut roses ‘All For Love’ and ‘White Beauty’ were used to compare cultivar-specific visible reflectance characteristics associated with contrasting petal pigmentation. HSI was performed at four time points, yielding 640 images per light source from 40 cut roses. The results revealed that the light source strongly affected both the image quality (mAP@0.5 60–80%) and VL (0–3 d) of cut roses. The HAL lamp produced high-quality spectral images across wavelengths (WL) ranging from 480 to 900 nm and yielded the highest object detection performance (ODP), reaching mAP@0.5 of 85% in ‘All For Love’ and 83% in ‘White Beauty’ with the YOLOv11x models. However, it increased petal temperature by 2.7–3 °C, thereby stimulating leaf transpiration and consequently shortening the VL of the flowers by 1–2.5 d. In contrast, INC produced unclear images with low spectral signals throughout the WL and consequently resulted in lower ODP, with mAP@0.5 of 74% and 69% in ‘All For Love’ and ‘White Beauty’, respectively. The INC only slightly increased petal temperature (1.2–1.3 °C) and shortened the VL by 1 d in the both cultivars. Although FLU and LED had only minor effects on petal temperature and VL, these illuminations generated transient spectral peaks in the WL range of 480–620 nm, resulting in decreased ODP (mAP@0.5 60–75%). Our results revealed that HAL provided reliable, high-quality spectral image data and high object detection accuracy, but simultaneously had negative effects on flower quality. Our findings suggest an alternative two-phase approach for illumination applications that uses HAL during the initial exploration of spectra corresponding to specific symptoms of interest, followed by LED for routine plant monitoring. Optimizing illumination in HSI will improve the accuracy of deep learning-based prediction and thereby contribute to the development of an automated quality sorting system that is urgently required in the cut flower industry.

## 1. Introduction

There has been a growing interest in the demand for reliable information and assurance of the quality of horticultural products as online commerce and sales continue to grow [1]. With the rapid development of artificial intelligence (AI) technology, studies on various camera techniques have been conducted to rapidly evaluate quality attributes, such as sugar content, physiological disorders, disease, and longevity of plants [2]. Among non-destructive camera-based detection techniques, hyperspectral imaging (HSI) has been widely investigated because it can capture extensive spectral and spatial information to evaluate physiological and stress conditions in many horticultural crops [3,4]. To obtain high-quality hyperspectral images (HSi), image acquisition conditions such as illumination, spatial resolution, and image sharpness must be carefully optimized [5]. In commercial HSI system, high spatial resolution is commonly achieved using line-scan systems, which require sequential acquisition and therefore increase total imaging time and the duration of sample exposure to the illuminant [6]. This constraint is critical for postharvest horticultural products because prolonged exposure can impose additional thermal or light stress that may alter water relations and accelerate senescence processes during imaging. Moreover, longer integration time is often adopted to improve signal-to-noise ratio (SNR), yet insufficient control of sensor noise and radiometric calibration can introduce spectral deviations that may be misinterpreted as stress or senescence-related signatures [7]. Because the sensors of hyperspectral cameras detect reflected light, the spectral nature of light sources, such as the lighting range and intensity, is a critical determinant of data quality [5,8].

Halogen (HAL) lamps have generally been used in HSI systems [5,8,9] because they provide a continuous spectral power distribution (SPD) and sufficient radiant output across the visible-near-infrared (VNIR) region for plant spectroscopy [9,10]. As thermal radiators, HAL sources also emit substantial energy at NIR and longer wavelengths, which increases radiative heat load at the sample plane during imaging. This heat load can raise tissue surface temperature and leaf-to-air vapor pressure deficit (VPD), thereby biasing transpiration-driven water relations in detached cut stems [9,11,12]. Such temperature-driven shifts may perturb gas exchange and accelerate water-stress symptoms, reducing postharvest quality in cut flowers [13,14]. Incandescent (INC) lamps operate on a similar thermal-emission principle [15,16], whereas light-emitting diodes (LED) and fluorescent (FLU) lamps generally show lower IR-related heat load but often have discontinuous SPD, which can cause band-specific signal deficits and spectral distortions in HSI [11,15,17,18].

Recently, attempts have been made to develop prediction models for cut flower longevity and disease development using HSI-based deep learning algorithms, and image-derived color features have been used to non-destructively estimate hydration-related quality loss [3,19,20]. Similarly, RGB image-extracted morphometric and color features combined with machine learning models have been used to estimate leaf area and SPAD value [21]. However, cut flowers, particularly cut roses, are perishable horticultural crops, and their longevity can change in response to abiotic stresses, such as high heat and light intensity, even over very short exposures [22,23]. Therefore, an appropriate illumination source should be used in the HSI system to maintain a good balance between high-quality data and minimal influence on plants. However, evidence remains limited on how HSI illumination conditions jointly affect spectral data quality and concurrent physiological responses in cut flowers.

Therefore, this study was conducted to systematically evaluate how different light sources (HAL, INC, FLU, and LED) affect the spectral and spatial quality of images, and to quantify the thermal load induced by each light source and its impact on the physiological state of cut roses. Specifically, we hypothesized that (i) HAL illumination imposes a higher thermal load during HSI acquisition, which elevates flower temperature, increases transpiration and water loss, and accelerates the deterioration of water relations, resulting in a shorter vase life (VL). We also expected that (ii) illumination providing more stable and uniform spectral signals improves hyperspectral input quality and thereby enhances object detection accuracy for flower buds, stems, and GMD lesions. Furthermore, we determined how these light-induced variations in spectral images directly influence the performance of object detection models tasked with identifying and localizing visual defects. In this study, a convolutional neural network (CNN) algorithm was used to build a deep learning-based object detection model, as it has been proven to be highly effective in extracting critical spectral signatures and spatial features required to assess plant health and disease status [24,25].

## 2. Results

### 2.1. Vase Life, Senescence Symptoms, and Postharvest Physiology

**Figure 1:**
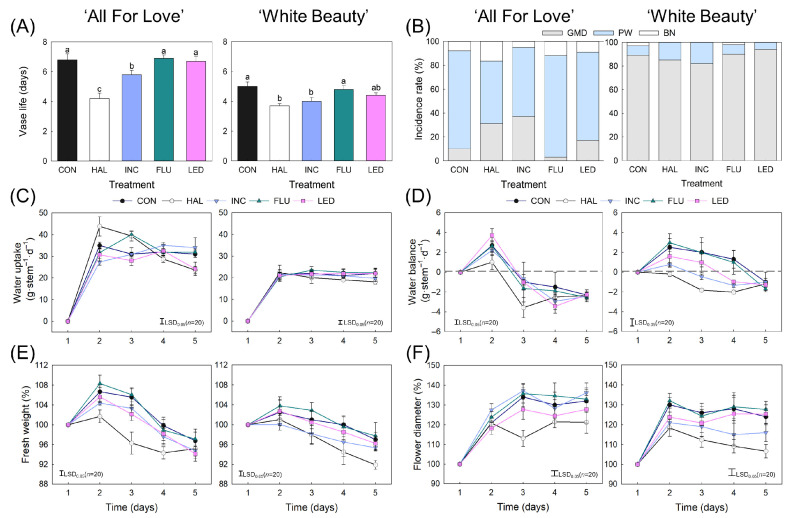
Effect of light sources on vase life (**A**), incidence rate of senescence symptoms (**B**) and postharvest quality factors (**C**–**F**) of cut roses ‘All For Love’ and ‘White Beauty’. Cut flowers were exposed to no light (CON) or to halogen (HAL), incandescent (INC), fluorescent (FLU), and light emitting diode (LED) lights for 60 s day^−1^ at before export simulation (BS), d 1, d 3, and d 6 of vase phase. GMD, gray mold disease; PW, petal wilting; BN, bent neck. Vertical bars indicate the standard error of the means (*n* = 20). The LSD bar in time course graph represents the least significant difference at *p* < 0.05. Different letters above the bars indicate significant differences according to Duncan’s multiple range test at *p* < 0.05.

The senescence and physiological characteristics of cut flowers changed significantly after exposure to various lighting conditions during HSI. HAL and INC reduced the VL of the cut flowers by 2.5 ± 0.4 d (*p* < 0.05) in ‘All For Love’ and by 1.2 ± 0.2 d (*p* < 0.05) in ‘White Beauty’, respectively, compared to the CON flowers. In contrast, FLU and LED had little to no impact on the VL (Figure 1A). The rapid VL reduction in ‘All For Love’ flowers was presumably due to their high susceptibility to water stress caused by the illumination. The predominant cause of VL termination was petal wilting (PW) in ‘All For Love’, whereas that was gray mold disease (GMD) in ‘White Beauty’. Exposure to HAL and INC increased the GMD ratio, whereas FLU increased PW compared to the CON flowers in ‘All For Love.’ Meanwhile, there was no significant change in the incidence rate at the VL termination following light exposure in ‘White Beauty’ (Figure 1B). In the ‘All For Love’ cultivar, HAL flowers showed the highest water uptake (WU) initially but exhibited a rapid decline from d 4 onward (Figure 1C). The WU reduction in the HAL flowers in ‘All For Love’ led to an early decrease in water balance (WB), fresh weight (FW) and flower diameter (FD) (Figure 1C–F). In ‘White Beauty,’ HAL and INC flowers showed an early collapse of the WB and subsequent decline in FD (Figure 1C–F). These results demonstrate that HAL and INC illumination negatively impacted postharvest quality and VL, whereas the influence of FLU and LED illumination on the flower quality of cut roses was negligible.

### 2.2. Petal Temperature, Transpiration, and Photosynthesis

**Figure 2:**
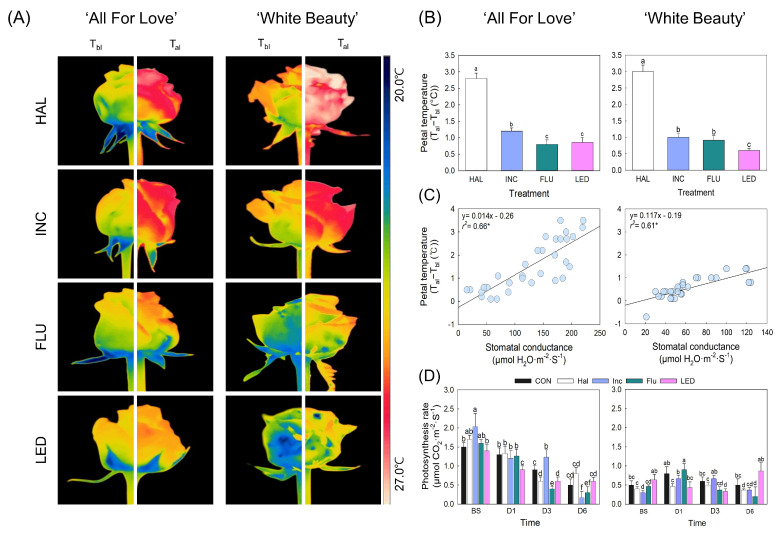
Effect of light source on changes in thermal images (**A**), petal temperature (**B**), petal temperature vs. stomatal conductance (**C**), and photosynthesis (**D**) of cut roses ‘All For Love’ and ‘White Beauty’. Cut flowers were exposed to no light (CON) or to halogen (HAL), incandescent (INC), fluorescent (FLU), and light emitting diode (LED) lights for 60 s day^−1^ at before export simulation (BS), d 1 (D1), d 3 (D3), and d 6 (D6) of vase phase. Temperature (T) of petals was calculated as T_al_ (T after lighting)—T_bl_ (T before lighting). Vertical bars indicate the standard error of the means (*n* = 6). Different letters above the bars indicate significant differences among all treatments at each time point as determined by Duncan’s multiple range test (*p* < 0.05).

The four light sources differentially affected petal temperatures in both cultivars (Figure 2). Petal temperature was highest in HAL, followed by INC in both cultivars (Figure 2A). Consistent with the thermal images, petal temperature was increased by more than 2.5 °C after HAL exposure, whereas it was minimally increased (≥1 °C) by LED (Figure 2B). Leaf transpiration also increased with increasing petal temperatures, as shown by the relatively high correlation between petal temperature and stomatal conductance in both cultivars in ‘All For Love’ (*r*^2^ = 0.66, *p* < 0.05) and in ‘White Beauty’ (*r*^2^ = 0.61, *p* < 0.05) (Figure 2C). However, light sources did not consistently affect leaf photosynthesis in cut roses. The photosynthetic rate was initially high before export simulation (BS) and then continually decreased toward the later stages in ‘All For Lover’, while it was generally low and showed no significant pattern among the treatments and times in ‘White Beauty’ (Figure 2D). The results indicated that increased temperature in flower buds induced by illumination stimulated leaf water loss; however, this did not affect leaf photosynthesis during the cut-flower phase.

### 2.3. Hyperspectral Imaging and Object Detection Performance

**Figure 3:**
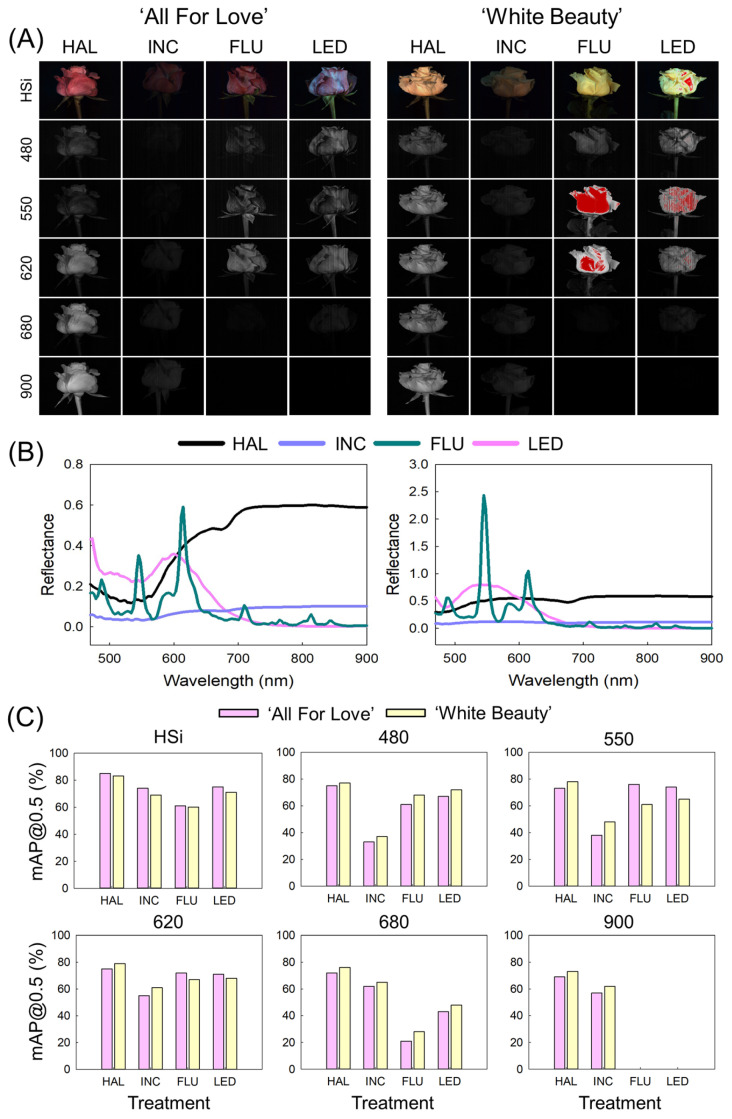
Effect of light sources on hyperspectral image (HSi) quality (**A**), reflectance spectrum (**B**); and object detection performance (**C**) of YOLO11x models in cut roses ‘All For Love’ and ‘White Beauty’. Cut flowers were exposed to no light (CON) or to halogen (HAL), incandescent (INC), fluorescent (FLU), and light emitting diode (LED) lights for 60 s day^−1^ at before export simulation (BS), d 1, d 3, and d 6 of vase phase. The photograph images were generated based on HSi and single band images (SBi) at 480, 550, 620, 680, and 900 nm. YOLOv11x model was trained using HSi and SBi within 480 to 900 nm for object detection of the flower buds. The mAP@0.5 indicates the evaluation index of the detection accuracy of the object detection models. The RS lines are average of 16 replicates (*n* = 16).

The light source in the HSI affected image quality, reflectance spectrum (RS), and object detection performance (ODP) of YOLO11x models (Figure 3). HAL generated a clear HSi and a single-band image (SBi) with low noise and high sharpness in the visible NIR (Vis/NIR) range (470–900 nm), whereas INC generated dark images with a low signal across the WL (Figure 3A). FLU and LED generated HSi and SBi (at 480, 550, and 620 nm, respectively) with slight noise, but they could not produce SBi in the WL at 680 and 900 nm. Consistently, SNR values measured from bud ROIs were highest under HAL (62 ± 0.3 dB), followed by INC (32 ± 2.1 dB), FLU (26 ± 2.8 dB), and LED (17 ± 3.3 dB) (Table S1). In ‘White Beauty’, FLU and LED produced saturation artifacts on flower buds at 550 and 620 nm, and the LED images exhibited vertical striping artifacts at 480, 550, and 620 nm (Figure 3A).

The difference in image quality is directly reflected in the RS of the flower buds. HAL showed the highest and most continuous RS across the entire WL range (480–900 nm) in both cultivars, whereas INC maintained low RS across the entire WL range (Figure 3B). Under FLU illumination, sharp and narrow peaks appeared at approximately 550 and 620 nm, which could explain the red saturation artifacts in the images (Figure 3A). LED produced distinct peaks in the blue and green regions and showed low RS in the red and NIR regions (Figure 3B). These results indicate that INC is not a suitable light source for image acquisition with the HSI system, and that FLU and LED cannot provide sufficient illumination for clear image acquisition in the NIR range.

The ODP (mAP@0.5) of the YOLO11x models differed among the image data acquired using different light sources (Figure 3C). HAL yielded the highest ODP in the HSi and SBi data for both cultivars. FLU resulted in the lowest ODP in HSi for both cultivars, while INC showed the lowest ODP for SBi 480, 550, and 620 nm. FLU and LED exhibited poor ODP in the NIR region (Figure 3C). A similar pattern was observed for mAP@0.5–0.9 (Figure S1). These results reveal that HAL is most effective under the tested conditions in obtaining high-quality HSi data and, consequently, high object detection accuracy based on HSi. FLU and LED lights may be used for HSI in some specific WL ranges (480–620 nm).

**Figure 4:**
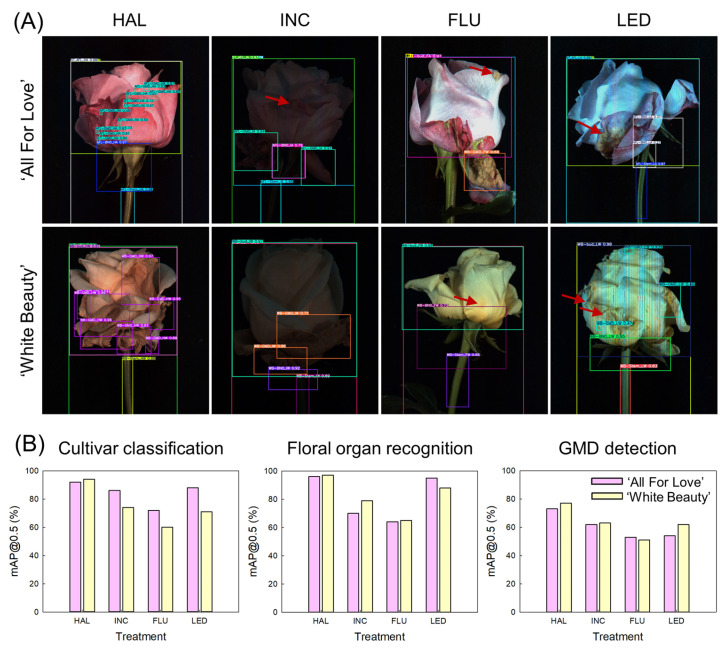
Object detection performance of YOLO11x for cut roses ‘All For Love’ and ‘White Beauty’. (**A**) Representative results of YOLO11x object detection based on hyperspectral image (HSi) obtained under different lighting sources. (**B**) Evaluation of the model performance in cultivar classification, floral organ recognition, and gray mold disease (GMD) detection in the cut rose cultivars. Cut flowers were exposed to halogen (HAL), incandescent (INC), fluorescent (FLU), and light emitting diode (LED) lights for 60 s day^−1^ at before export simulation (BS), d 1, d 3, and d 6 of vase phase. The YOLOv11x models were trained using HSi dataset. Red arrow indicates the GMD lesion that was not detected by the YOLO11x models. Bounding box shows the detected regions of various objects, such as cultivars, opening levels, stem condition, and wounded area and GMD lesion on petals. The mAP@0.5 indicates the evaluation index of the detection accuracy of the object detection models.

The ODP of the YOLOv11x model was evaluated for cultivar classification, floral organ recognition, and GMD detection in two cut rose cultivars using HSi datasets acquired under four different light conditions. The HAL model identified multiple objects in the images, including flower buds, stems, and GMD lesions (Figure 4A). In contrast, the INC, FLU, and LED models failed to clearly distinguish between floral organs and GMD lesions.

The HAL model yielded the highest mAP@0.5 values for identifying cultivars and detecting floral parts in both rose cultivars (Figure 4B). The LED model showed relatively high accuracy in detecting cultivars and floral organs in ‘All For Love,’ but the accuracy decreased in ‘White Beauty,’ indicating that the quality of HSi obtained under LED varied with flower type and color. The INC and FLU models had the lowest accuracy in detecting cultivars and floral organs, with wide variations between the rose cultivars. Compared with cultivar classification and floral organ detection, GMD detection showed markedly lower mAP@0.5 values across lighting sources (Figure 4B). The HAL model exhibited the highest performance in GMD detection, whereas the INC, FLU, and LED models showed a substantially lower ODP (Figure 4B). These results demonstrate that the light source strongly affects the data quality of HSi, thereby influencing the ODP of the YOLOv11x models. It was also revealed that model performance varied greatly depending on the detection targets in the images, such as flower color and disease area.

## 3. Discussion

Our results demonstrate that the properties of the illuminant profoundly impact not only data quality and model performance but also the physiological status of plants, particularly in cut roses, which are vulnerable to abiotic stresses. The HAL lamp increased the temperature of the floral organs and, consequently, caused excessive water loss from the leaves, resulting in early deterioration of the water balance. In cut roses with limited water and carbohydrate supply even small temperature increases can accelerate water balance collapse [26,27]. It was expected that the petal temperature increased by HAL would increase photosynthesis by stimulating stomatal opening [5]; however, its effect was limited to the induction of transpiration in cut rose flowers. The rapid collapse of the water balance leads to early petal wilting, subsequently shortening the VL of cut roses [28]. The contrasting cultivar responses likely reflect genotype-dependent differences in postharvest water relations and in disease susceptibility. Cultivar variability in VL is strongly influenced by water loss control and the ability to maintain water balance when environmental load increases, and rose cultivars can differ in transpiration behavior and stomatal responsiveness [29]. In contrast to HAL, LED and FLU produced negligible thermal load and had minimal impact on the physiology of cut flowers, suggesting that the use of these light sources in HSI may better preserve postharvest quality of cut flowers compared to HAL lighting. This interpretation is consistent with the concurrent increases in petal and leaf temperature during hyperspectral acquisition and with the positive relationship between petal temperature and stomatal conductance (Figure 2C) which preceded the earlier decline in water balance under HAL (Figure 1) [30]. To clarify how the short illumination-induced heat is related to senescence, the downstream stress signaling pathway should be addressed in future work.

In contrast, during image acquisition with a hyperspectral camera, HAL lamps generated the clearest spectral images across the WL, consistent with previous studies showing the high-energy emission of HAL lamps in the Vis/NIR regions [5]. In contrast, FLU and LED lamps produced clear images only within limited WL (480–620 nm) in colored cultivars, such as ‘All For Lover,’ and otherwise only in narrow visible WL (480 nm). YOLOv11x models based on HSi acquired under Hal illumination generated the highest mean average precision for both cultivar classification and flower bud detection. Among the light sources, HAL was the only illuminant that allowed reliable detection of GMD lesions. The LED lamp provided high performance as a HAL for analyzing simple visible features, but it failed to identify objects in the WL at 900 nm. These results indicate that illumination affects not only the raw image quality but also the ultimate machine vision accuracy of the image data. However, model performance should be interpreted cautiously because GMD lesions were less frequent than cultivar and floral-organ labels which can bias learning toward dominant classes even with a stem-level stratified split [31]. In addition, differences in spectra across light sources and vase stages may reduce generalization, so the reported accuracy mainly reflects performance under the current dataset and imaging setup and should be confirmed using independent data from other cultivars and acquisition conditions [32].

Taken together, the Hal lamp provides the broadest spectral coverage and the highest accuracy of object detection; however, its strong heat is significantly detrimental to the quality and longevity of cut flowers. Therefore, HAL lamps should be used in HSI systems when the goal is to explore specific spectral features of plants. Once a key diagnostic band is identified, the illuminant can be switched to other light sources, such as LED, which operate effectively in specific WL regions while generating low heat. Properly designed LED arrays would deliver adequate signals in the visible WL region without imposing a thermal load [17,33]. Previous studies have also shown that uniformly diffused light is essential for HSI to avoid hotspots on plants and ensure spectral fidelity in 3D images [5,11,33]. Our results highlight a practical trade-off between spectral performance and thermal load across illuminants, and we therefore propose a two-phase workflow as a testable hypothesis. In an initial setup step, HAL with a light diffuser may be used to support wavelength selection and system calibration, including evaluation of bands in the WL region. In a subsequent step, LED illumination may be more suitable for routine monitoring to limit additional thermal stress during repeated imaging; however, this integrated two-phase workflow was not directly tested in the present study and should be validated in a dedicated experiment.

Although our current study clearly evaluated the impact of light sources on the quality of spectral data and cut flowers, there are still additional investigations that should be conducted. This study showed the effects of lighting properties in two rose cultivars that have distinct differences in color and senescence patterns. However, it is necessary to examine spectral changes and heat damage across a larger number of cultivars with different pigment profiles and across cut-flower species with different petal morphology, and to confirm performance under commercial packing-line settings to extend this system setup beyond the two cultivars tested. The LED lamp used in this study also had limited broadband output in the visible region and provided weak signals toward the red to NIR region; thus, alternative broadband lamps or mixed LED arrays covering wider wavelength ranges should be examined.

This study compared illumination sources that are commonly used among commercial products to evaluate potential utilization of the illumination for the HSI system. Thus, incomplete irradiance matching is acknowledged as a limitation when interpreting cross-source differences in signal level and perceived image quality. A limitation of this study is that full radiometric calibration was not available to report absolute irradiance at the sample plane in W·m^−2^ and to compute exposure dose in radiometric units. In our experiment, the heating pattern was not proportional to nominal wattage, as HAL bulb (20 W) produced the higher temperature increase than INC bulb that has higher-wattage (25 W). In the next experimental phase, we will measure spectral irradiance using a calibrated spectroradiometer under the same imaging geometry to report W·m^−2^ and energy dose per exposure and to enable intensity-normalized comparisons by equalizing irradiance or photon flux at the sample plane ideally by wavelength band.

The floricultural industry faces a growing need for automated sorting and classification of product quality to facilitate the growth of e-commerce markets by providing reliable information on product value to consumers. Light stimuli can modulate postharvest quality in ornamentals, so light source choices can affect both measurement fidelity and biological responses during imaging [34]. The performance of HSI-based systems can be compromised by technical factors such as the choice of a light source. The results of this study demonstrate that the high analytical performance of HAL comes at the cost of physiological damage, especially in cut flowers, highlighting the importance of balancing spectral adequacy and thermal load of light sources. Our study suggests an alternative two-phase HSI system combining HAL illumination during the initial setup phase and LED illumination during the routine monitoring phase to simultaneously obtain spectral fidelity with thermal safety. In practical packing line use, a single-scan workflow with short acquisition time is required to meet throughput targets while minimizing heat accumulation, and illumination should be selected with consideration of energy use and thermal risk to cut flower quality. LED is generally more energy efficient and easier to manage thermally than HAL, whereas HAL may increase cooling demand and thermal risk during repeated or prolonged scanning. Transferability should be validated across cultivars and cut-flower species with different pigment profiles and morphologies, and robust deployment may require periodic recalibration and model updating under site-specific hardware and illumination conditions. The optimized illumination system in HSI would not only improve the reliability of quality assessment based on a deep learning prediction model but also provide economic and energy-efficiency benefits throughout the supply chain, ultimately contributing to the stability and growth of the cut flower industry.

## 4. Materials and Methods

### 4.1. Plant Material

Cut rose cultivars (*Rosa hybrida* L. ‘All For Love’ and ‘White Beauty’) were obtained from a commercial greenhouse in Gokseong, Korea. The rose plants were transplanted on rockwool slabs in a greenhouse in March 2022 and drip-irrigated with a nutrient solution [0.97 mM Ca(NO_3_)_2_·4H_2_O, 2.5 mM NH_4_NO_3_, 0.09 mM KNO_3_, 1.502 mM MgSO_4_·7H_2_O, 0.67 mM KH_2_PO_4_, and trace concentrations of micronutrients]. Roses were harvested at the commercial half-open stage [35,36,37] in February 2023. The cut flowers were transported to the laboratory at Gyeongkuk National University, Andong, Korea, for 3 h in tap water. Upon arrival, the cut flowers underwent HSI under various light sources and were subsequently kept under a controlled environment (10 ± 1 °C and RH 50 ± 5% in the dark) for three days for export simulation to Japan [38].

### 4.2. Vase Life Assessment

After the export simulation, the flower stems were recut to a length of 45 cm, retaining the three uppermost leaves, and placed individually in a vase containing 450 mL of distilled water held in an environmental controlled room (23 ± 1 °C, 50 ± 2% RH, and a 12 h photoperiod). For VL evaluation, 20 cut roses per cultivar, meeting used replication levels for cut roses VL experiments, were monitored daily for FW, WU, VL, and senescence [39]. FD was measured daily using a digital caliper (CD-20APX, Mitutoyo Corporation, Kanagawa, Japan) to track the opening process, and WB was calculated from FW and WU. VL was defined as the number of days until a flower was considered senescent. The termination of VL was defined by the appearance of one or more of the following symptoms: pedicel bending (BN, with an angle > 45° from vertical), PW (loss of turgor in ≥ 50% of the petals), or GMD (necrotic lesions affected ≥ 50% of petal area). The primary symptoms that caused VL termination were recorded for each cut flower [36].

### 4.3. Hyperspectral Imaging and Illumination Treatments

**Figure 5:**
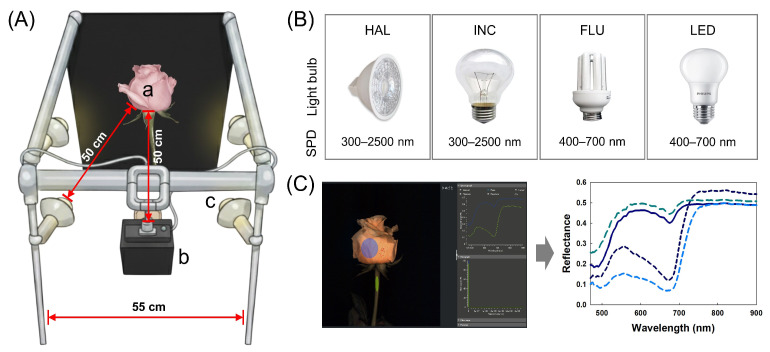
Experimental setup for hyperspectral imaging (HSI) system. (**A**) Configuration of the HSI system; (**B**) Light bulb types and their spectral power distribution (SPD); (**C**) Data analysis process showing the selected region of interest (ROI; blue and green circle) on the flower bud (**left**) and the extraction of corresponding reflectance spectra (**right**). HAL, halogen; INC, incandescent; FLU, fluorescent; and LED, light emitting diode. The red arrows indicate the working distance between the subject (a) and the hyperspectral camera (b) or light bulbs (c) (50 cm) and the spacing between the light bulbs (c) (55 cm). In (**C**), the blue and light green circles on the right indicate example ROIs selected for the flower bud and stem, respectively; reflectance spectra extracted from each ROI can be visualized as shown in the left example.

HSI was conducted on 40 cut flowers four times: just before the export simulation (BS) and days 1, 3 and 6 (D1, D3, and D6) of the vase phase. The cut flowers were imaged sequentially under four different illuminations using a Vis/NIR line hyperspectral camera (SnapScan; IMEC, Leuven, Belgium). The camera featured a sensor resolution of 3650 × 2048 pixels and captured 150 spectral bands across the 470–900 nm range. Each cut flower was imaged from two opposite viewing angles (0° and 180°) by rotating the flowers (Figure 5A) and all images were captured in reflectance mode under controlled conditions (23 ± 2 °C, 50 ± 2% RH). This imaging schedule yielded 640 images per light source.

The lighting system used for HSI consisted of four lamps mounted symmetrically at a 45° angle to the optical axis and positioned 50 cm away from the subject (Figure 5A). This fixed 45° symmetric illumination geometry was used for all treatments to minimize angle dependent variability in reflectance and to isolate the effect of illuminant type under a standardized configuration [40,41]. The light sources examined in this study were HAL (4 × 20 W halogen lamps, OSRAM, Munich, Germany; correlated color temperature (CCT) = 2800 K), FLU (4 × 20 W Osram compact fluorescent lamps, OSRAM, Munich, Germany; CCT = 4000 K), LED (4 × 20 W Philips LED lamps, Philips, Amsterdam, Netherlands; CCT = 6500 K), and INC (4 × 25 W Ilkwang incandescent lamps, Ilkwang, Daegu, Korea; CCT = 2700 K) (Figure 5B). All lamps were newly purchased for this experiment, and illuminant-specific white and dark references were acquired whenever the illuminant was switched to minimize temporal drift effects on reflectance calibration. All light bulbs were prewarmed for 15 min to stabilize their output, and each scan was recorded with a camera integration time of 2 ms. Illuminance at the sample plane was measured for each light source using a calibrated lux meter (CANA-0010, Tokyo Photoelectric, Tokyo, Japan). The measured illuminance at the flower bud was 37,400 lx for HAL, 1895 lx for FLU, 28,400 lx for LED, and 338 lx for INC. For each illuminant (and whenever the illuminant was switched), a white reference panel (95% reflectance) and a dark-reference frame (SnapScan internal shutter) were acquired under the same geometry, and raw data were converted to reflectance using the corresponding illuminant-specific references in IMEC SnapScan software v1.8.1 (IMEC, Leuven, Belgium) to generate calibrated reflectance hypercubes for each cut flower and treatment (Figure 5C). SNR was used to quantify radiometric noise under each illumination condition. A homogeneous region of interest (ROI) was defined on the flower bud surface, and band-wise SNR was calculated as SNR = μROI/σROI, where μROI is the mean reflectance and σROI is the standard deviation of pixel values within the ROI.

### 4.4. Thermal Imaging and Temperature Analysis

The surface temperature of each flower was nondestructively detected using a thermograph (passive infrared thermal camera; FLIR T530, FLIR Systems, Wilsonville, OR, USA), positioned 45 cm from the subject. The thermography, which operates in the long-wave infrared (LWIR, 7.5–14 μm) range, captured thermal images (radiometric jpg format). We set thermal emissivity to 0.98 for rose petal tissues for all thermal analyses and applied the same setting consistently across treatments [42,43]. For accurate temperature readings, a blackbody reference plate (10 × 10 cm, 2 mm thick, emissivity of 0.97) was placed next to the flower. All thermal images were acquired under the same controlled conditions as the HSI (23 ± 2 °C, 50 ± 2% RH).

Each HSi acquisition (at BS, D1, D3, and D6) was bracketed with two thermal images captured immediately before and after the scan to quantify the heating effect of the light sources. The acquired thermal images were analyzed using FLIR Tools software version 5.7 (FLIR Systems) to extract the average petal surface temperature (T_p_). The temperature of the reference plate (T_r_) was determined to calculate the normalized differential temperature (T_p_ − T_r_). To quantify the heating effect of illumination during HSI, the change in the petal temperature was calculated as the difference between the temperatures after (T_al_ = (T_p_ − T_r_)—after light) and before (T_bl_ = (T_p_ − T_r_)—before light) illumination (T_al_ − T_bl_).

### 4.5. Photosynthesis and Stomatal Conductance Measurements

Leaf gas exchange parameters, including net photosynthetic rate and stomatal conductance, were measured immediately following imaging acquisitions at BS, D1, D3, and D6. These measurements were included to assess whether brief HSI illumination induces changes in leaf gas exchange that are associated with transpiration-driven water balance during the vase period. Measurements were taken from the uppermost, fully expanded leaves of six stems per cultivar (*n* = 6). Replication was limited to six stems per cultivar because each gas-exchange measurement required stabilization in the cuvette and was completed within a consistent short post-imaging time window to minimize time-dependent physiological drift. The measurements were performed using a portable infrared gas analysis system (CIRAS-3, PP Systems, Amesbury, MA, USA) equipped with a leaf cuvette (4.5 cm^2^ window). The conditions inside the cuvette were maintained at a photosynthetic photon flux density (PPFD) of 50 μmol·m^−2^·s^−1^, leaf temperature of 20 °C, reference CO_2_ concentration of 380 μmol·mol^−1^, and 50% RH to ensure a constant vapor pressure deficit (VPD) [44,45]. Stomatal conductance was determined based on water vapor measurements simultaneously with the net photosynthetic rate after the readings had reached a steady state. The instrument was calibrated before each measurement session.

### 4.6. Object Detection Model

The dataset used in this study comprised 2560 images generated from HSI. The objects of interest for detection were the flower buds, stems, and GMD lesions. The flowers were treated as image-level classes and encoded as a single bounding box encompassing the main inflorescence. Quality degradation factors were annotated with bounding boxes by a single expert familiar with rose quality and disease symptoms using the LabelMe (version 1.8.1, MIT, MA, USA). Annotations were initially drawn as rectangles and then converted to the YOLO format (class, x-center, y-center, width, and height). To ensure consistency, all annotations were reviewed in a second pass by the same annotator prior to conversion to YOLO format.

The HSI data were processed into HSi and SBi as the model input. HSi was generated by combining all SBi from 470 to 900 nm into an RGB rendering. For SBi, images from the 487-, 680-, and 900 nm bands were used as single-channel inputs. All original images were then letterboxed to a resolution of 640 × 640 pixels while preserving the aspect ratio, and the pixel values were scaled to the range of [0, 1]. Letterboxing was applied only to the 2D HSi/SBi images after reflectance conversion to match the fixed YOLO input size using aspect-ratio-preserving resize with padding, and it was not applied to the calibrated hyperspectral cubes used for reflectance-spectrum analysis. The dataset was partitioned into training (70%), validation (20%), and test (10%) sets using a stem-level stratified split to prevent data leakage (1792, 512, and 256 images, respectively).

The YOLOv11x model (Ultralytics, Frederick, MD, USA) was used for object detection and implemented in PyTorch (version 2.0.1 + cu118, Linux Foundation, San Francisco, USA). The model was trained on a system equipped with an Intel i9-13900K CPU and NVIDIA RTX 4090 GPUs (2 × 24 GB). The training hyperparameters included an input size of 640 × 640, batch size of 16, and 200 epochs. The conservative data augmentation strategy, employed to maintain spectral fidelity, included random horizontal flip (*p* = 0.5), random affine transformations (scale ± 10%, rotation ± 5°), moderate brightness and contrast adjustments (±10%), and mild Gaussian noise (σ ≤ 5/255). Additive Gaussian noise was applied only to the HSi and SBi after reflectance calibration to emulate radiometric sensor noise and improve robustness, and it was not used for RS analysis. Spectral bias was prevented by disabling hue-based augmentation for single-band inputs and limiting it to color-composite images.

Model inference utilized a confidence threshold of 0.25 and a non-maximum suppression (NMS) Intersection over Union threshold of 0.70. The performance of the model on the test set was evaluated using mAP@0.5 and mAP@0.5–0.9. All reported detection metrics were computed on the test set (256 images) under the study imaging conditions. First, the utility of specific spectral bands was assessed by training the models independently and comparing detection performance across the four input data streams. Second, using color-composite images, performance was analyzed for three specific tasks: cultivar classification, flower bud detection, and GMD detection.

### 4.7. Statistical Analysis

Data are presented as the mean ± standard error (SE) of 20 biological replicates per treatment (*n* = 20). Statistical significance (*p* < 0.05) was determined using one-way or two-way analysis of variance (ANOVA), followed by Duncan’s multiple range test for post hoc comparisons, where appropriate. These analyses were conducted using SPSS software (version 22.0; IBM, Somers, NY, USA).

## Data Availability

Data will be made available on request.

## Supplementary Materials

The following supporting information can be downloaded at: https://www.mdpi.com/article/10.3390/plants15020215/s1, Supplementary Table S1: SNR of hyperspectral images acquired under different illumination sources in two cut rose cultivars (‘All For Love’ and ‘White Beauty’); Figure S1: Effect of light sources on hyperspectral image (HSi) quality in cut roses ‘All For Love’ and ‘White Beauty’.

## References

[1] Hassoun A., Kamiloglu S., Garcia-Garcia G., Parra-López C., Trollman H., Jagtap S., Aadil R.M., Esatbeyoglu T. (2023). Implementation of relevant fourth industrial revolution innovations across the supply chain of fruits and vegetables: A short update on Traceability 4.0. Food Chem..

[2] Wieme J., Mollazade K., Malounas I., Zude-Sasse M., Zhao M., Gowen A., Argyropoulos D., Fountas S., Van Beek J. (2022). Application of hyperspectral imaging systems and artificial intelligence for quality assessment of fruit, vegetables and mushrooms: A review. Biosyst. Eng..

[3] Zerdoner M., Gabriëls S., Arens P., Visser R.G., Mishra P. (2025). Detection of *Botrytis cinerea* severity in rose petals using hyperspectral imaging for plant breeding applications. Comput. Electron. Agric..

[4] Choi S.Y., Lee A.K. (2020). Development of a cut rose longevity prediction model using thermography and machine learning. Hortic. Sci. Technol..

[5] Detring J., Barreto A., Mahlein A.-K., Paulus S. (2024). Quality assurance of hyperspectral imaging systems for neural network supported plant phenotyping. Plant Methods.

[6] Qin J., Kim M.S., Chao K., Chan D.E., Delwiche S.R., Cho B.-K. (2017). Line-scan hyperspectral imaging techniques for food safety and quality applications. Appl. Sci..

[7] Paulus S., Mahlein A.-K. (2020). Technical workflows for hyperspectral plant image assessment and processing on the greenhouse and laboratory scale. Gigascience.

[8] Gómez-Sanchís J., Moltó E., Camps-Valls G., Gómez-Chova L., Aleixos N., Blasco J. (2008). Automatic correction of the effects of the light source on spherical objects. An application to the analysis of hyperspectral images of citrus fruits. J. Food Eng..

[9] Fu X., Wang X., Rao X. (2017). An LED-based spectrally tuneable light source for visible and near-infrared spectroscopy analysis: A case study for sugar content estimation of citrus. Biosyst. Eng..

[10] Lee H., Cho S., Lim J., Lee A., Kim G., Song D.-J., Chun S.-W., Kim M.-J., Mo C. (2023). Performance comparison of tungsten-halogen light and phosphor-converted NIR LED in soluble solid content estimation of apple. Sensors.

[11] Qin J., Monje O., Nugent M.R., Finn J.R., O’Rourke A.E., Wilson K.D., Fritsche R.F., Baek I., Chan D.E., Kim M.S. (2023). A hyperspectral plant health monitoring system for space crop production. Front. Plant Sci..

[12] Appeltans S., Guerrero A., Nawar S., Pieters J., Mouazen A.M. (2020). Practical recommendations for hyperspectral and thermal proximal disease sensing in potato and leek fields. Remote Sens..

[13] Hüve K., Bichele I., Kaldmäe H., Rasulov B., Valladares F., Niinemets Ü. (2019). Responses of aspen leaves to heatflecks: Both damaging and non-damaging rapid temperature excursions reduce photosynthesis. Plants.

[14] Moore C.E., Meacham-Hensold K., Lemonnier P., Slattery R.A., Benjamin C., Bernacchi C.J., Lawson T., Cavanagh A.P. (2021). The effect of increasing temperature on crop photosynthesis: From enzymes to ecosystems. J. Exp. Bot..

[15] Elvidge C.D., Keith D.M., Tuttle B.T., Baugh K.E. (2010). Spectral identification of lighting type and character. Sensors.

[16] Azizi M., Golmohammadi R., Aliabadi M. (2016). Comparative analysis of lighting characteristics and ultraviolet emissions from commercial compact fluorescent and incandescent lamps. J. Res. Health Sci..

[17] Stergar J., Hren R., Milanič M. (2022). Design and validation of a custom-made laboratory hyperspectral imaging system for biomedical applications using a broadband LED light source. Sensors.

[18] Mahlein A.-K., Hammersley S., Oerke E.-C., Dehne H.-W., Goldbach H., Grieve B. (2015). Supplemental blue LED lighting array to improve the signal quality in hyperspectral imaging of plants. Sensors.

[19] Kim S., Lee A. (2024). Predicting blooming day of cut lily through wavelength reflectance analysis. Horticulturae.

[20] Makraki T., Tsaniklidis G., Papadimitriou D.M., Taheri-Garavand A., Fanourakis D. (2025). Non-Destructive monitoring of postharvest hydration in cucumber fruit using Visible-Light color analysis and Machine-Learning models. Horticulturae.

[21] Tsaniklidis G., Makraki T., Papadimitriou D., Nikoloudakis N., Taheri-Garavand A., Fanourakis D. (2025). Non-destructive estimation of area and greenness in leaf and seedling scales: A case study in cucumber. Agronomy.

[22] Ha S.T.T., Nguyen T.K., Lim J.H. (2021). Effects of air-exposure time on water relations, longevity, and aquaporin-related gene expression of cut roses. Hortic. Environ. Biotechnol..

[23] Evelyn S., Farrell A.D., Elibox W., De Abreu K., Umaharan P. (2020). The impact of light on vase life in (*Anthurium andraeanum* Hort.) cut flowers. Postharvest Biol. Technol..

[24] Ou Y., Yan J., Liang Z., Zhang B. (2024). Hyperspectral Imaging Combined with Deep Learning for the Early Detection of Strawberry Leaf Gray Mold Disease. Agronomy.

[25] Yan T., Xu W., Lin J., Duan L., Gao P., Zhang C., Lv X. (2021). Combining multi-dimensional convolutional neural network (CNN) with visualization method for detection of *Aphis gossypii* Glover infection in cotton leaves using hyperspectral imaging. Front. Plant Sci..

[26] Fanourakis D., Papadakis V.M., Psyllakis E., Tzanakakis V.A., Nektarios P.A. (2022). The role of water relations and oxidative stress in the vase life response to prolonged storage: A case study in chrysanthemum. Agriculture.

[27] Verdonk J.C., van Ieperen W., Carvalho D.R., van Geest G., Schouten R.E. (2023). Effect of preharvest conditions on cut-flower quality. Front. Plant Sci..

[28] Lear B., Casey M., Stead A.D., Rogers H.J. (2022). Peduncle necking in Rosa hybrida induces stress-related transcription factors, upregulates galactose metabolism, and downregulates phenylpropanoid biosynthesis genes. Front. Plant Sci..

[29] Fanourakis D., Carvalho S.M., Almeida D.P., van Kooten O., van Doorn W.G., Heuvelink E. (2012). Postharvest water relations in cut rose cultivars with contrasting sensitivity to high relative air humidity during growth. Postharvest Biol. Technol..

[30] Vialet-Chabrand S., Lawson T. (2019). Dynamic leaf energy balance: Deriving stomatal conductance from thermal imaging in a dynamic environment. J. Exp. Bot..

[31] Miftahushudur T., Sahin H.M., Grieve B., Yin H. (2025). A survey of methods for addressing imbalance data problems in agriculture applications. Remote Sens..

[32] Li B., Ma T., Inagaki T., Tsuchikawa S. (2025). Enhanced detection of early bruises in apples using near-infrared hyperspectral imaging with geometrical influence correction for universal size adaptation. Postharvest Biol. Technol..

[33] Song J.-Y., Bian L.-f., Sun X.-m., Ding Z., Yang C. (2022). Design of active hyperspectral light source based on compact light pipe with LED deflection layout. Opt. Laser Technol..

[34] Horibe T. (2020). Use of light stimuli as a postharvest technology for cut flowers. Front. Plant Sci..

[35] Harkema H., Mensink M.G., Somhorst D.P., Pedreschi R.P., Westra E.H. (2013). Reduction of *Botrytis cinerea* incidence in cut roses (*Rosa hybrida* L.) during long term transport in dry conditions. Postharvest Biol. Technol..

[36] VBN (2014). Evaluation Cards for Rosa.

[37] Shi L., He S., Wang Z., Kim W.S. (2021). Influence of nocturnal supplemental lighting and different irrigation regimes on vase life and vase performance of the hybrid rose ‘Charming Black’. Hortic. Sci. Technol..

[38] In B.-C., Lee J.-H., Lee A.-K., Lim J.H. (2016). Conditions during export affect the potential vase life of cut roses (*Rosa hybrida* L.). Hortic. Environ. Biotechnol..

[39] Macnish A.J., Leonard R.T., Borda A.M., Nell T.A. (2010). Genotypic variation in the postharvest performance and ethylene sensitivity of cut rose flowers. Hortscience.

[40] Cooksey C.C., Allen D.W., Tsai B.K., Yoon H.W. (2015). Establishment and application of the 0/45 reflectance factor scale over the shortwave infrared. Appl. Opt..

[41] Bodner G., Nakhforoosh A., Arnold T., Leitner D. (2018). Hyperspectral imaging: A novel approach for plant root phenotyping. Plant Methods.

[42] Chen C. (2015). Determining the leaf emissivity of three crops by infrared thermometry. Sensors.

[43] López A., Molina-Aiz F., Valera D., Peña A. (2012). Determining the emissivity of the leaves of nine horticultural crops by means of infrared thermography. Sci. Hortic..

[44] Kang C., Zhang Y., Cheng R., Kaiser E., Yang Q., Li T. (2021). Acclimating cucumber plants to blue supplemental light promotes growth in full sunlight. Front. Plant Sci..

[45] Costa L., Montano Y.M., Carrión C., Rolny N., Guiamet J.J. (2013). Application of low intensity light pulses to delay postharvest senescence of *Ocimum basilicum* leaves. Postharvest Biol. Technol..

