# Supplementary material for: Light Sources in Hyperspectral Imaging Simultaneously Influence Object Detection Performance and Vase Life of Cut Roses
Source: Plants (Basel). 2026 Jan 9;15(2):215. doi: 10.3390/plants15020215 (PMC12844643; doi:10.3390/plants15020215)
Supplement: Supplementary file 1 [file plants-15-00215-s001.zip › plants-4062489-supplementary.pdf]

Supplementary Material

Supplementary Tables

Table S1. SNR of hyperspectral images acquired under different illumination sources in two cut rose cultivars ('All For Love' and 'White Beauty').

Cultivars	HAL	INC	FLU	LED
**'All For Love'**	62 ± 0.3 dB	32 ± 2.1 dB	26 ± 2.8 dB	17 ± 3.3 dB
**'White Beauty'**	63 ± 0.2 dB	37 ± 1.2 dB	29 ± 2.3 dB	19 ± 4.5 dB

Supplementary Figures

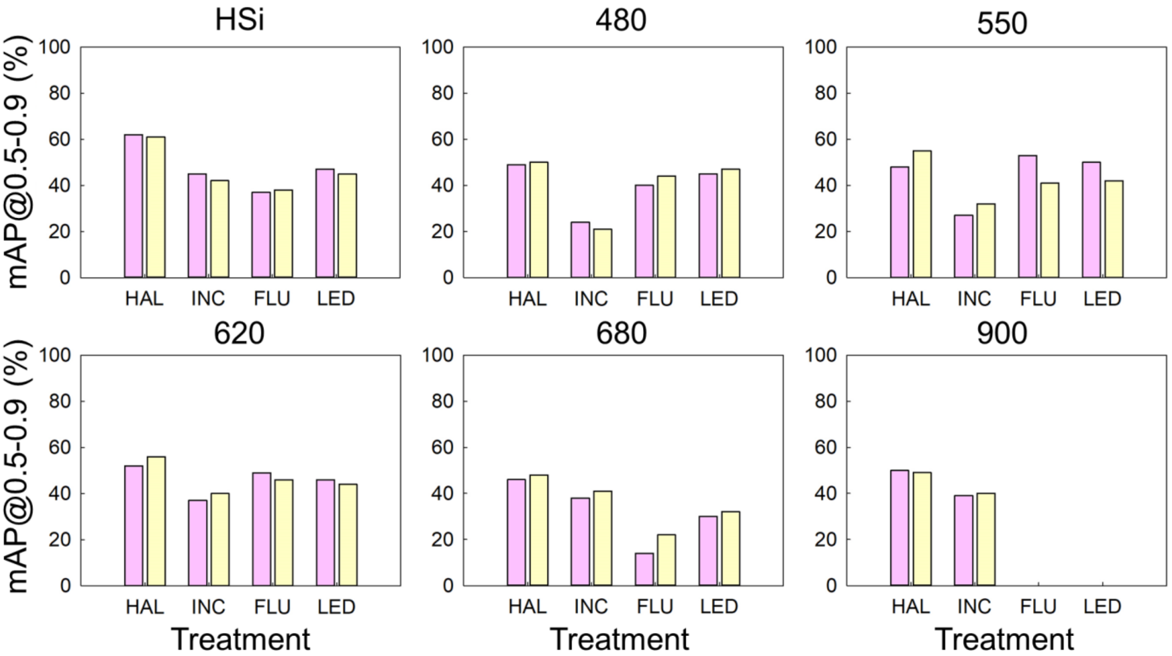

Figure S1. Effect of light sources on hyperspectral image (HSi) quality in cut roses 'All For Love' and 'White Beauty'. Cut flowers were exposed to no light (CON) or to halogen (HAL), incandescent (INC), fluorescent (FLU), and light emitting diode (LED) lights for 60 sec day-1 at before export simulation (BS), d 1, d 3, and d 6 of vase phase. The photograph images were generated based on HSi and single band images (SBi) at 480, 550, 620, 680, and 900 nm. YOLOv11x model was trained using HSi and SBi within 480 to 900 nm for object detection of the flower buds. The mAP@0.5 indicates

the evaluation index of the detection accuracy of the object detection models.
